# Hydrogen-Free Plasma Nitriding Process for Fabrication of Expanded Austenite Layer on AISI 316 Stainless Steel Surface

**DOI:** 10.3390/ma18010140

**Published:** 2025-01-01

**Authors:** Mitsuhiro Hirano, Koyo Miura, Naofumi Ohtsu

**Affiliations:** Faculty of Engineering, Kitami Institute of Technology, 165 Koen-cho, Kitami 090-8507, Hokkaido, Japannohtsu@mail.kitami-it.ac.jp (N.O.)

**Keywords:** hydrogen-free plasma nitriding, austenitic stainless steel, nitrogen-expanded austenitic phase, N_2_-Ar gas mixture, radio frequency discharge mode, etching mitigation

## Abstract

The addition of hydrogen to nitrogen facilitates the formation of nitride phases in the plasma nitriding processes of stainless steels, though it also induces the deterioration of their mechanical properties. This study presents a hydrogen-free plasma nitriding process for fabricating a nitrogen-expanded austenite phase (γ_N_) on an AISI 316 stainless steel surface. The steel substrate was nitrided in N_2_-Ar plasma with various gas compositions discharged by radio frequency (RF) and direct current (DC) modes. The process using the RF mode enabled the fabrication of a layer composed of a γ_N_ phase with a thickness of approximately 3 μm on the steel surface regardless of the gas composition, thereby enhancing its surface hardness. In contrast, such a layer was not observed in the DC mode, and the steel’s hardness was similar to that of the untreated surface. This difference in layer formation was attributed to the mitigation of surface etching by the Ar active species using the RF mode because of the lower bias voltage compared with that of the DC mode. This phenomenon suppresses the removal of the nitride phase formed during the process, which is a key factor contributing to nitrogen penetration. In conclusion, an N_2_-Ar plasma nitriding process using the RF mode is demonstrated to be a hydrogen-free process for fabricating a layer of a γ_N_ phase.

## 1. Introduction

Plasma nitriding using a glow discharge enables the diffusion of nitrogen atoms to the interstitial sites of a metal surface via exposure to nitrogen (N_2_) plasma for at least several hours. For austenitic stainless steels, a metastable nitride phase called the expanded austenite phase (γ_N_) is formed on the surfaces [1,2,3]. The layer composed of the γN phase enhances the wear resistance through an increase in the surface hardness because of the residual compressive stresses induced by the penetration of nitrogen atoms [4,5,6,7]. In stainless steel, the penetration of nitrogen atoms is impeded by a thin passive oxide layer formed on the surface. Thus, the oxide layer must be removed simultaneously while nitriding the surface.

Conventionally, mixing hydrogen (H_2_) gas with N_2_ gas has been used as an effective approach. The atomic hydrogen contained in the discharged plasma acts as a chemical etching agent for removing the passive layer [8,9] and simultaneously induces lattice strain because of the interfacial solution in the steel matrix [10,11]. These phenomena facilitate the penetration of nitrogen atoms, thereby improving the plasma nitriding efficiency of stainless steels [11,12,13]. However, the penetration and accumulation of hydrogen atoms cause hydrogen embrittlement, leading to deterioration of the mechanical strength of the metallic substrate owing to hydrogen-induced cracking [14,15,16,17,18,19,20,21]. Therefore, an alternative gas mixture that does not require H_2_ must be investigated to develop a hydrogen-free plasma nitriding process.

Argon (Ar) gas is a possible candidate for replacing H_2_ gas in the nitriding process. Physical etching with Ar active species, such as its ions in the plasma, enables the removal of the oxide layer owing to the sputtering effect [22], which has been applied as a pretreatment to enhance nitriding efficiency. Previously, we demonstrated that the penetration of nitrogen atoms into the AISI 316 surface was facilitated by sequential treatment using pure Ar and N_2_ plasma generated via the direct current (DC) mode [11]. Furthermore, Abrasonis et al. reported that Ar^+^ ion irradiation using a Kaufman-type ion source for 1 min on the AISI 304 L surface before the nitriding process increased the amount and depth of nitrogen penetration [23]. However, mixing Ar with N_2_ gas impeded the formation of the nitride phase. In our previous study, the AISI 316 surface was treated using DC-discharged N_2_-Ar plasma with a gas composition of 30–90% Ar, whereas the layer composed of the γ_N_ phase was not fabricated, regardless of the composition [11]. This is because the nitride phase was removed immediately from the N_2_-Ar plasma by performing etching together with nitriding, suppressing nitrogen penetration.

This unfavorable phenomenon was probably caused by excessive etching of the Ar active species. To address this problem, we focused on the discharge mode for generating N_2_-Ar plasma. The bias voltage of the radio frequency (RF) discharged plasma is approximately half that of the DC plasma [24], implying that the kinematic energy of the Ar active species is inferior. The etching rate on a material surface achieved using the Ar plasma-generated RF discharge mode is higher than that obtained using DC plasma [25,26,27]. Based on these characteristics, we conjecture that applying the RF discharge mode can suppress etching and, consequently, enhance nitrogen penetration in the N_2_-Ar plasma.

Herein, we aim to fabricate a layer comprising a γ_N_ phase on a stainless steel substrate via the N_2_-Ar plasma nitriding process using the RF discharge mode without H_2_ gas. To this end, an AISI 316 stainless steel (316SS) specimen was nitrided in plasma discharged by gas mixtures comprising N_2_ and Ar in various ratios using laboratory-made apparatus. The resultant surface and plasma characteristics were analyzed, and the effect of the discharge mode on the layer formation was investigated and compared with that obtained using the DC discharge mode. Finally, the industrial applicability of the nitriding process was discussed.

## 2. Materials and Methods

### 2.1. Specimen Preparation

**Figure 1:**
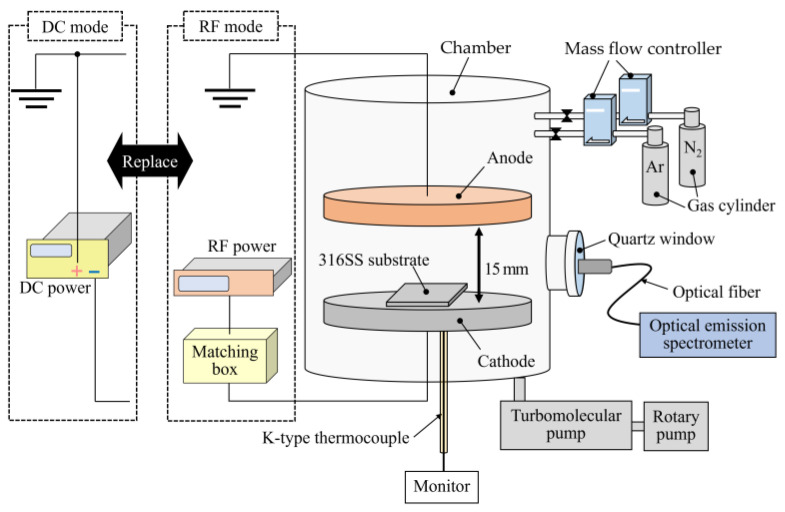
Schematic diagram of plasma nitriding apparatus with RF and DC discharge modes.

Plasma nitriding was performed using apparatus built in our laboratory, as shown in Figure 1. The cathode and anode were composed of stainless steel and copper disks, respectively, located parallel to each other in the chamber at a distance of 15 mm. The anode was connected to the ground. A 13.56 MHz RF generator with an automatic impedance-matching unit and a DC power supply was utilized to generate plasma. The pressure in the chamber was evaluated as ~5.0 × 10^−4^ Pa using a rotary pump and a turbomolecular pump. Discharge gases were introduced from individual gas inlet lines attached to the chamber, and their flow speeds were adjusted using mass flow controllers. An optical emission spectrometer (HR4000, Ocean Optics, Orlando, FL, USA) was fixed to a synthetic quartz window using an optical fiber to obtain the plasma emission spectra. Furthermore, a K-type thermocouple was set to the back of the cathode to monitor the surface temperature.

**Figure 2:**
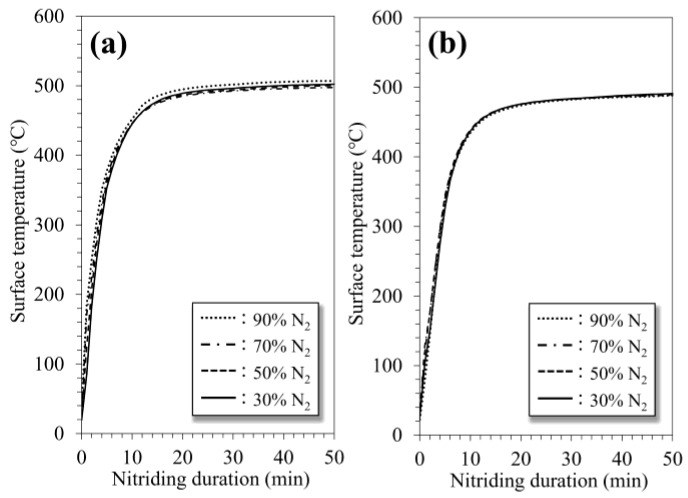
Variations in substrate surface temperature against nitriding duration: (**a**) RF glow discharge and (**b**) DC glow discharge mode.

316SS (Nilaco Co., Tokyo, Japan) with dimensions of 10 × 10 × 1 mm^3^ was used as the substrate. Silicon carbide paper and colloidal silica suspensions were used to give the substrates a mirror-like finish. The specimens were sonicated in ethanol for 10 min. Subsequently, they were placed at the center of the cathode, and the chamber was evacuated. N_2_ and Ar gases were introduced from their respective inlets, and the gas composition was adjusted to 10–70% Ar by controlling the flow. Hereafter, the specimens are labeled based on the composition of the Ar gas; for example, 30% Ar. The RF glow discharge was performed at a constant pressure of 30 Pa in the chamber while applying a constant power of 100 W. For the DC glow discharge, the pressure was maintained at 300 Pa, after which it was discharged at a constant current of 0.1 A. The applied DC voltage monotonically increased from 320 V through the treatment, eventually reaching less than 600 V. Thus, the process using the DC mode was performed within 30–60 W. The gas pressure and electrical conditions for generating the plasma were determined based on prior measurements of the optical emission intensity to prevent a drastic difference in the population of Ar active species between the discharge modes. The substrates were treated with the plasma generated by each discharge mode for 50 min without auxiliary substrate heating. The surface temperature reached approximately 500 °C, regardless of the discharge mode and gas composition (Figure 2). Finally, the substrates were cooled naturally to room temperature under a vacuum at 10^−4^ Pa level.

### 2.2. Surface Analysis

The crystal phase of the surface was determined using X-ray diffraction (XRD; D8 ADVANCE, Bruker AXS, Karlsruhe, Germany) with Cu Kα radiation using Bragg–Brentano geometry. Cross-sectional morphological images were obtained using scanning electron microscopy (SEM; JCM-5000 NeoScope, JEOL, Tokyo, Japan) at an acceleration voltage of 10 kV in the secondary electron image mode. Mirror-like polishing and chemical etching with Marble’s solution were used to prepare the cross sections of the specimens. The hardness of the plasma-treated surface was evaluated using a Vickers hardness tester (HMV-G; Shimadzu, Kyoto, Japan) under a load of 0.49 N, corresponding to 50 gf. Measurements were performed at seven points on the surface.

## 3. Results

### 3.1. Characteristics of 316SS Surface Nitrided in N_2_-Ar Plasma Discharged by RF and DC Modes

**Figure 3:**
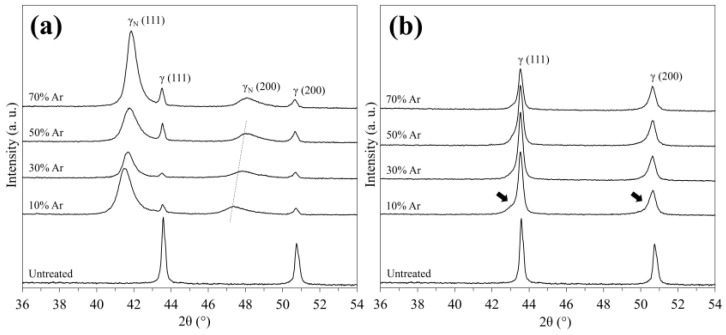
XRD patterns of 316SS surface treated in N_2_-Ar plasmas: (**a**) RF glow discharge and (**b**) DC glow discharge mode.

The XRD patterns of the untreated surfaces and the 316SS surface nitrided in the N_2_-Ar plasma generated using RF glow discharge are shown in Figure 3a. Two major peaks corresponding to γ-Fe (111) and (200) were determined on the untreated surface; thus, the substrate was a single austenite phase. New peaks appeared on the pattern of the 10% Ar surface treated using RF-discharged plasma at a lower diffraction angle when compared with γ-Fe peaks. These peaks are attributed to the austenite phase expanded by the interfacial solute nitrogen atom (γ_N_) [11,28], indicating that the layer composing the γ_N_ phase was fabricated via plasma nitriding. Furthermore, the γ_N_ (200) peak was monotonically shifted to a higher angle through an increase in the composition of Ar. It has been reported that the degree of peak shift reflecting the lattice expansion correlates with the nitrogen content dissolved in the surface layer [29]. We demonstrated that the shift to a lower angle of approximately 47° obtained for a nitride 316 SS surface corresponded to a nitrogen content of approximately 5 mass% [11]. Therefore, the variations suggest that the maximum nitrogen content in the layer fabricated on the 10% Ar surface was 5 mass%, which decreased with increasing Ar gas composition. In contrast, the peaks attributed to the γ_N_ phase were not detected on the XRD pattern of the surface treated using the DC-discharged plasma, regardless of the gas composition (Figure 3b). The γ-Fe (111) and (200) peaks were broadened to a lower diffraction angle (indicated by black arrows). The broadened region contracts with increasing Ar gas content. This peak was associated with the induction of lattice strain, indicating that plasma nitriding dissolved nitrogen atoms interstitially in the γ-Fe lattice [11]. Based on these XRD results, we conclude that the penetration and diffusion of nitrogen atoms were facilitated in the plasma discharged in the RF glow discharge mode. Furthermore, an increase in Ar content suppressed the penetration of atoms.

**Figure 4:**
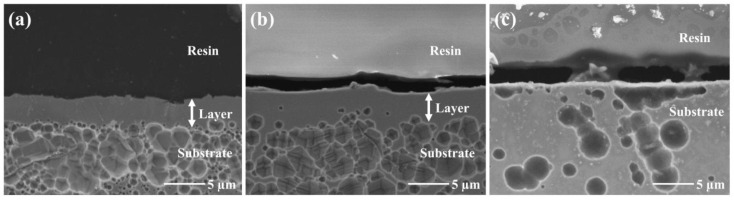
Cross-sectional SEM images of (**a**) 10% Ar and (**b**) 70% Ar surfaces treated in N_2_-Ar plasma generated using RF discharge and (**c**) 70% Ar surface treated by DC discharge.

Cross-sectional SEM images of typical nitrided specimens are shown in Figure 4. The SEM observations of the 10% Ar surface treated with the RF glow-discharged plasma demonstrate the formation of a homogeneous layer with a thickness of approximately 3.5 μm (Figure 4a). This result implies that this surface layer is a nitride layer composed of the γ_N_ phase identified using the XRD analysis. Hardly any significant difference in thickness was observed among the specimens prepared in the RF discharge mode and, thus, their thickness seems to be independent of the gas composition (Figure 4a,b). In contrast, no surface layer was observed in the SEM images of the substrate that was nitrided using the DC glow discharge, as shown in Figure 4c.

### 3.2. Vickers Hardness of Nitrided 316SS Surface

**Figure 5:**
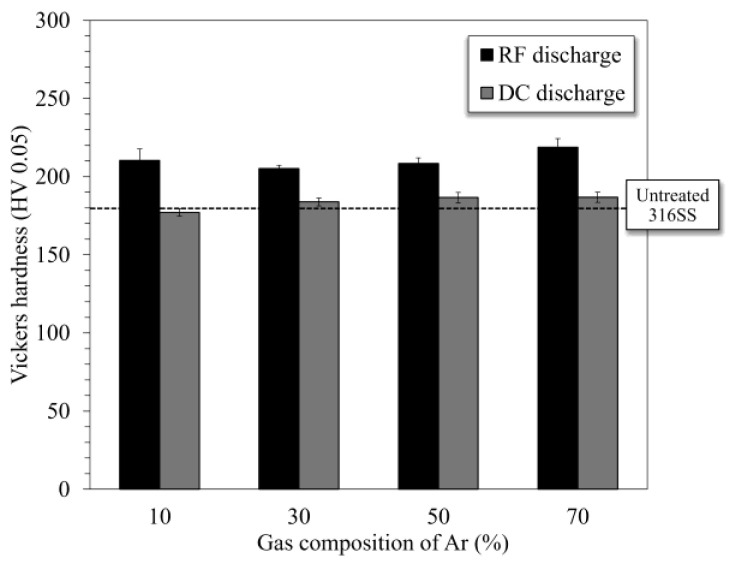
Vickers hardness of 316SS surfaces nitrided in N_2_-Ar plasmas generated using RF and DC glow discharge modes.

The Vickers hardness values of the nitrided 316SS surfaces are shown in Figure 5. The hardness of the surface nitrided via RF discharge increased compared to that of the untreated surface, reaching approximately 220 HV, regardless of the gas composition. However, no obvious differences were found between the hardness values of the untreated and DC samples. Therefore, plasma nitriding using RF glow discharge is suggested to enhance hardness owing to the fabrication of the nitride layer comprising the γ_N_ phase.

### 3.3. Plasma Diagnosis of RF and DC Glow-Discharged N_2_-Ar Plasma

**Figure 6:**
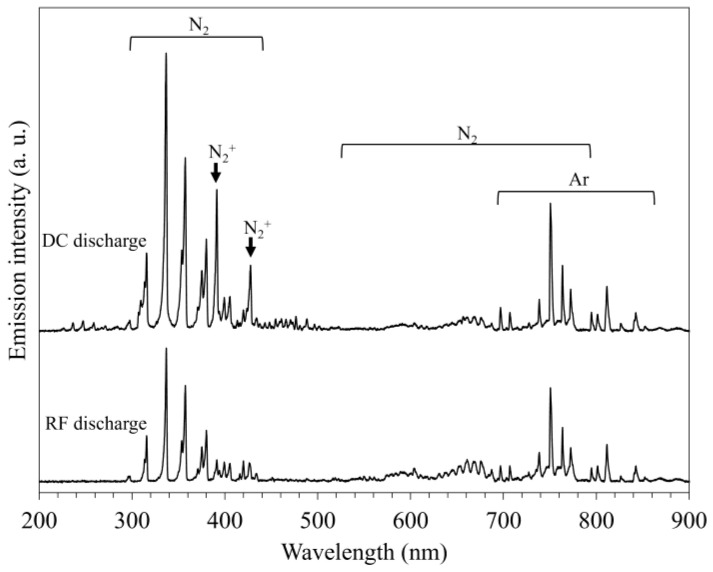
Typical optical emission spectra of N_2_-Ar plasmas corresponding to 50% Ar conditions generated using RF and DC glow discharge modes.

To understand the difference when fabricating the layer comprising the γ_N_ phase in the plasma discharge mode, the active species in the plasma were determined by measuring the emission spectra of the N_2_-Ar plasma. The typical emission spectra of the N_2_-Ar plasma generated by the RF and DC glow discharges using a gas mixture corresponding to 50% Ar are shown in Figure 6. These spectra comprise emission lines and bands corresponding to excited N_2_ in the ranges of 300–450 and 500–800 nm [30,31]. Furthermore, major lines attributed to nitrogen molecular ions (N_2_^+^) were observed at 391.4 and 427.8 nm [30]. Intense lines corresponding to excited Ar were found in the 600–850 nm range [32,33].

**Figure 7:**
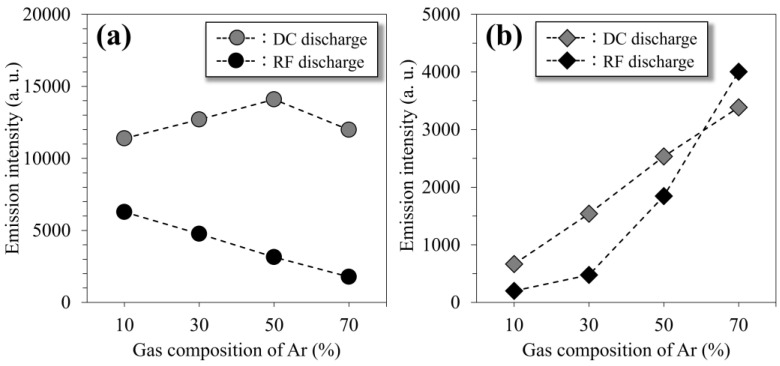
Emission intensities attributed to (**a**) N_2_^+^ (391.4 nm) and (**b**) excited Ar (811.4 nm), plotted against gas composition of Ar.

The N_2_^+^ and Ar active species are the principal factors related to the progress of the nitriding reaction [34] and the sputtering effect, respectively. The variation in the emission line intensities correlated to these populations with different gas compositions is shown in Figure 6. The intensity of N_2_^+^ (391.4 nm) in the spectra obtained by the RF discharge decreased monotonically with an increase in the Ar gas composition (Figure 7a). The intensity of the DC discharge increased slightly, up to 50% Ar, whereas it was higher than that of the RF discharge regardless of the gas composition. The intensity of the excited Ar (811.4 nm) increased with an increase in the Ar gas composition, and no drastic difference was found between the RF and DC discharges (Figure 7b). Thus, these results demonstrate that the population of N_2_^+^ species was higher in the DC glow-discharged plasma, whereas that of the Ar active species was similar between the N_2_-Ar plasmas generated using the two glow discharge modes.

## 4. Discussion

### 4.1. Effect of Discharge Mode on Nitrogen Penetration During N_2_-Ar Plasma Nitriding Process

Characterization of the nitrided 316SS surface indicated that the discharge mode used in the N_2_-Ar plasma nitriding process dominated the fabrication of the γ_N_ phase. Accordingly, the penetration of nitrogen atoms was facilitated in the plasma generated in the RF glow discharge mode, whereas its progress was suppressed in the DC glow discharge mode. These results are probably caused by differences in the plasma’s characteristics and/or surface reactions during the nitriding process.

Previous studies have reported that the population of active species and the substrate temperature are key factors promoting the nitriding reaction [4,11,34]. The population of N_2_^+^ species affects the facilitation of the nitriding reaction. In the present study, the population was higher in the DC glow-discharged plasma owing to the emission intensity, regardless of the gas composition (Figure 7a). In addition, no noticeable difference existed in the populations of the Ar species contributing to physical etching between the discharge modes (Figure 7b). The substrate temperature dominated the thermal diffusion of nitrogen atoms into the substrate; however, no difference was observed between the discharge modes (Figure 2). These results indicate that the DC glow discharge mode provides a more appropriate nitriding atmosphere for the fabrication of the nitride phase compared to the RF mode, whereas the penetration of nitrogen atoms is not facilitated. Therefore, these parameters do not play a critical role in the penetration of nitrogen during the N_2_-Ar plasma nitriding process.

The bias voltage is another factor that dominates the kinetic energy of the active species and is correlated with the progress of physical etching. Pérez et al. investigated the correlation between sputtering rate and applied power by exposing metallic substrates to RF and DC glow-discharged plasmas [25]. They demonstrated that the RF glow discharge mode requires 4 to 8 times higher power than the DC discharge mode to achieve the same sputtering rate; thus, the kinetic energy of the Ar active species—that is, the bias voltage—in the RF mode is inferior. We performed plasma nitriding in the RF mode at a power of 100 W, which was approximately 1.6 times higher than that of the DC mode. Therefore, the bias voltage was inferior in the RF mode under nitriding conditions, indicating that the etching of the 316SS surface was mitigated.

**Figure 8:**
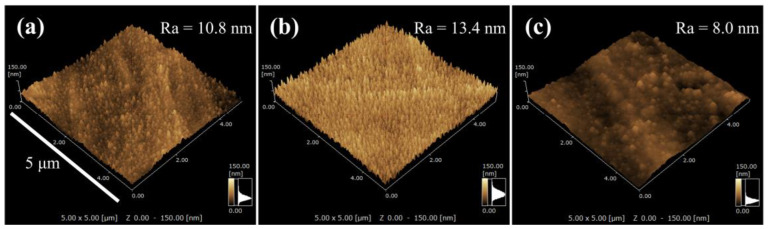
SPM images of 316SS surfaces nitrided in N_2_-Ar plasmas generated by RF (**a**,**b**) and DC glow discharge modes (**c**): (**a**) 10% Ar, (**b**,**c**) 70% Ar.

To confirm the impact of the proposed discharge mode on the progress of etching, the typical surface morphologies of the plasma-nitrided 316SS were observed using a scanning probe microscope (SPM), as shown in Figure 8. Numerous nanoprotrusions were observed on the entire 10% Ar surface nitrided in the RF glow-discharged plasma. Their height and number density appeared to increase with increasing Ar gas composition (Figure 8a,b). In contrast, the 70% Ar surface nitrided in the DC glow-discharged plasma was smoother, and the nanogranules appeared sparse (Figure 8c). The average roughness (Ra) values calculated from the SPM images were 10.8, 13.4, and 8.0 nm for the 10% Ar and 70% Ar surfaces nitrided by the RF plasma and the 70% Ar surface treated by the DC plasma, respectively. Furthermore, the value of the RF discharge increased slightly with increasing Ar gas composition, and it was higher than that of the DC discharge regardless of the gas composition. Our previous study demonstrated that the presence of nanoprotrusions provides evidence attributable to the progression of etching on the 316SS surface. In contrast, excessive etching at higher bias voltages induces the appearance of nanogranules owing to the decay of nanopillars, resulting in the formation of a smooth surface [35]. These SPM observations suggest that the removal of the nitride phase was suppressed by the utilization of the RF discharge mode due to mildly progressing physical etching, which promoted the penetration and diffusion of nitrogen atoms. This phenomenon is a key event in the fabrication of nitrided layers via the N_2_-Ar plasma nitriding process. However, an increase in the gas composition of Ar enhanced the etching; thus, the fabrication of the nitride phase was impeded. This result was supported by the peak’s shift to a higher diffraction angle in the XRD pattern (Figure 3a). Hence, we conclude that N_2_-Ar plasma RF-discharged with a gas mixture containing small amounts of Ar favors the fabrication of the layer comprising the γ_N_ phase on a 316SS surface.

### 4.2. Industrial Application of N_2_-Ar Plasma Nitriding Process Employing RF Discharge Mode

In this study, the N_2_-Ar plasma nitriding process employing the RF discharge mode enabled the fabrication of a layer composed of the γ_N_ phase on a 316SS surface without H_2_ gas. We expect that this process would be applicable to other austenitic stainless steels with higher hydrogen embrittlement susceptibility, such as AISI 301 and 304, and would probably prevent the deterioration of mechanical properties induced by hydrogen embrittlement, and consequently fractures. However, the surface hardness of the layer must be improved for the industrial application of the proposed nitriding process. Although the surface hardness of the layer was increased to approximately 220 HV via the N_2_-Ar plasma nitriding process, a surface hardness of at least 650 HV is required to improve the wear resistance when fabricating the γ_N_ phase, according to previous studies [7,36,37,38]. To overcome this problem, the penetration of nitrogen into the layer must be further improved to reinforce the lattice expansion of the steel surface. In the present study, the gas composition of Ar was correlated with the degree of expansion (Figure 3a); this was likely promoted by employing an admixture ratio of less than 10% Ar, owing to the optimization of physical etching. Furthermore, other parameters such as processing duration, gas pressure, and applied power are also important for the penetration of nitrogen atoms. To improve the hydrogen-free nitriding process, our research group is focusing on the aforementioned conditions.

## 5. Conclusions

This study demonstrates that a N_2_-Ar plasma nitriding process using the RF discharge mode fabricated a layer comprising a γ_N_ phase with a thickness of approximately 3 μm on a 316SS surface, enhancing the surface’s hardness. However, no layer was found when the process was performed using the DC discharge mode. From the resultant surfaces, it was suggested that the layer formation is dominated by the progress of etching with the Ar active species. The RF discharge mode mitigated the removal of the nitride phase by suppressing physical etching, owing to its lower bias voltage compared to the DC mode, thereby promoting nitrogen penetration and diffusion. We believe that these findings provide a hydrogen-free nitriding process applicable to austenitic stainless steels with high hydrogen embrittlement susceptibility. However, further improvement and optimization of the nitriding conditions is necessary for its industrial application.

## Data Availability

The original contributions presented in this study are included in the article. Further inquiries can be directed to the corresponding author.
